# Surveillance of Human Cases of Salmonellosis, Campylobacteriosis, Listeriosis, and Hepatitis A in Campania (Southern Italy): Seven-Year Monitoring (2013–2019)

**DOI:** 10.3390/pathogens12010048

**Published:** 2022-12-28

**Authors:** Germana Colarusso, Maria Francesca Peruzy, Polina Mazzone, Rosa Luisa Ambrosio, Roberta Pellicanò, Angelo D’Argenzio, Aniello Anastasio, Loredana Baldi

**Affiliations:** 1 Epidemiology and Biostatistics Coordination Department, Experimental Zooprophylactic Institute of Southern, Via Salute 2, 80055 Portici, Italy; 2 Department of Veterinary Medicine and Animal Production, University of Naples Federico II, 80137 Napoli, Italy; 3 UOD Prevention and Sanitary Hygiene—Prevention and Protection of Health and Safety in Living and Working Environments–O.E.R., Campania Region, 80143 Napoli, Italy

**Keywords:** foodborne diseases, surveillance system, salmonellosis, campylobacteriosis, listeriosis, hepatitis A

## Abstract

Foodborne infections cause illness and death every year worldwide. The aim of this study was to describe trends in 2013–2019 in the occurrence of human cases of salmonellosis, campylobacteriosis, listeriosis, and hepatitis A in the Campania region. Human case data were provided by the National Surveillance System of disease and were grouped by year, province, age group, and sex. Moreover, the number of people hospitalized was recorded. In the Campania region, the total number of confirmed human cases for the diseases investigated was 1924, with Hepatitis A and the Salmonellosis as the first most reported (1009 and 825 cases, respectively). The incidence rates of gastroenteritis under study were lower than those in Italy and European Union in the same period, with the exception of Hepatitis A whose incidence was higher than that recorded in Italy. Data on hospitalizations pointed out the onset of severe forms of infection also for listeriosis and campylobacteriosis, whose incidence was very low (27 and 63 cases, respectively). Unfortunately, no information on the foods implicated is available. Although probably underestimated, gastroenteritis due to foodborne agents still represents a burden in Campania, and continuous monitoring and implementation of the currently available regional surveillance system is required.

## 1. Introduction

Foodborne disease (FBD) is an illness caused by the consumption of food or beverage contaminated by pathogenic bacteria or their toxins, viruses, or other microorganisms [1]. Foodborne diseases causing acute gastroenteritis are a major problem worldwide [2]. However, FBDs range from mild, self-limiting illness to more serious illness, such as kidney or liver failure, brain, and neural disorders, and even death [3]. People at risk from more serious illnesses are children, pregnant women, elderly people, and immunocompromised persons [4].

In 2015, the World Health Organization (WHO) published the world’s first estimates of the global and regional incidence and burden of FBDs. This research estimated that in 2010, 31 major foodborne hazards resulted in over 600 million illnesses and 420,000 deaths worldwide [5]. FBDs cause around 33 million global disability-adjusted life year (DALY) losses each year and therefore have a negative impact on the economy of a country [6]. Even though the burden per 100,000 habitants is higher in low-income countries, the economic impact due to FBDs on health services is considerable in the high-income nations [7]. According to the U.S. Centers for Disease Control and Prevention (CDC) around 48 million people are hospitalized, and 3000 die each year [7]. In 2010 in the European Region, according to a report published in 2017 by WHO, more than 23 million people fell ill from eating contaminated food, resulting in an estimated 4654 deaths with the diarrhoeal disease agents as the most frequent causes of foodborne illness [8]. The WHO estimates show that in the European region, the burden due to the bacteria *Salmonella* spp., *Campylobacter* spp., and *Listeria monocytogenes* and the viruses Norovirus and hepatitis A is of particular concern [8]. The number of confirmed cases reported by EFSA and ECDC in Italy between 2013 and 2019 ranged from 3256 (in 2019) to 5048 (in 2013) for salmonellosis, from 1014 (in 2015) to 1633 (in 2019) for campylobacteriosis, from 132 (in 2014) to 202 (in 2019) for listeriosis, and from 487 (in 2015) to 3766 (in 2017) for hepatitis A (http://atlas.ecdc.europa.eu/public/index.aspx, accessed on 5 December 2022).

However, the number of human cases, and therefore the true incidence of the disease, is probably underestimated. Underestimation is likely a combination of underdiagnosis and under-reporting due to the characteristics of each reporting system and because most cases do not require medical care [9]. 

In the European Union (EU), data collection for burden analysis of foodborne diseases is managed through a pyramidal system, in which member states must provide the European Food Safety Authority (EFSA) with all data collected on zoonoses, foodborne outbreaks, and antimicrobial resistance (Directive 2003/99/EC), including cases of salmonellosis, campylobacteriosis, and listeriosis. Concerning hepatitis A, the notification is mandatory based on the current epidemiological situation. 

In Italy, the Ministerial Decree (DM) 15/12/1990 regulates the notification system of infectious disease based on their categorization in five classes by importance and impact on public health, and surveillance is part of the activities of the National Surveillance System of disease (PREMAL). In particular, notifications of confirmed cases are transferred from primary care or self-employed physicians, laboratories and hospital physicians to regional health authorities and then to the Ministry of Health, which hosts the PREMAL. From one step to another, all information must be validated within 60 days to advance to the next level. In PREMAL the following data fields are available: patient personal data (e.g., name, age, sex), health data, physician data, additional recipients of the report, additional information (e.g., specific documentation for the different diseases), clinical, epidemiological and laboratory information. 

Besides PREMAL, pathogen surveillance is also carried out by voluntary surveillance systems such as Enter-Net that collects data of confirmed cases for different enteric bacterial pathogens (such as *Listeria*, *Salmonella*, and *Campylobacter*) and the Integrated Epidemiological System of Acute Viral Hepatitis (SEIEVA). 

The importance of studying the burden of Foodborne Diseases in each country has recently been recognized, mainly due to the lack of homogeneous investigative resources and data to estimate it equally on a global scale [10]. Although standardization of notification has been fixed, the management of foodborne outbreaks does not follow national protocols, and the task is entrusted to the individual regional authorities, which operate by applying their operational guidelines. Therefore, although an overview of the foodborne disease in human is published each year by EFSA and ECDC, data on the infection occurring in each region are missing. To this purpose, a clearer view of the trend of specific gastrointestinal (GI) infections in each Italian region could be useful in defining policy approaches and public health priorities [11]. The present work aimed to provide an overview of the incidence of salmonellosis, campylobacteriosis, listeriosis, and hepatitis A in the Campania region, the most populous region of southern Italy (ISTAT, 2021; http://dati.istat.it, accessed on 21 April 2022), and their distribution between the five provinces (Avellino, Benevento, Caserta, Napoli, and Salerno) using official notification data collected in 2013–2019.

## 2. Materials and Methods

### 2.1. Data Collection

Notification data from 2013 to 2019 for salmonellosis, campylobacteriosis, listeriosis, and hepatitis A in the Campania region were obtained from the PREMAL online database. This collects data of confirmed human cases from primary care and self-employed physicians, laboratories and hospital physicians. Data on the diseases under investigation were collected per year, age group (0–4 years, 5–9 years, 10–14 years, 15–24 years, 25–44 years, 45–64 years, and >64 years), sex, and province (Avellino, AV; Benevento, BN; Caserta, CE; Napoli, NA; Salerno, SA). Moreover, the number of people hospitalized was also recorded.

### 2.2. Data Analysis

Incidence data analysis was performed using SPSS version 27 (IBM Analytics, Armonk, NY, USA). The incidence rates of each gastroenteritis event by year in Campania Region were measured as the number of new cases of disease per 100,000 individuals calculated on the whole population residing in the region between 2013 and 2019 (https://demo.istat.it, accessed on 21 April 2022). These data were compared to those reported in EFSA/ECDC reports related to Italian and European population (http://atlas.ecdc.europa.eu/public/index.aspx, accessed on 16 March 2022). Moreover, sex and age-specific incidences were calculated for each disease on the specific category of population (female and male, and 0–4 years, 5–14, 15–24, 25–44, 45–64, 65 years and older, respectively) residing in the Campania Region between 2013 to 2019 (https://demo.istat.it, accessed on 21 April 2022). ANOVA one-way and t-test analyses were carried out to statistically analyse the differences in incidence rates among mean annual incidences of age-specific groups and genders, respectively. Statistical significance was determined at *p* < 0.05. 

The geographical distribution of notifications per each disease in the Campania region was mapped using QGIS software version 3.22.3. The geographical references from 2013 to 2019 relating to the patients’ residence were transferred to the QGIS system and the total number of confirmed cases per each gastroenteritis event was calculated at the province level (Avellino, Benevento, Caserta, Napoli and Salerno). The cases were geo-referenced considering the home as the most likely place of origin of the foodborne illness.

## 3. Results

**Figure 1:**
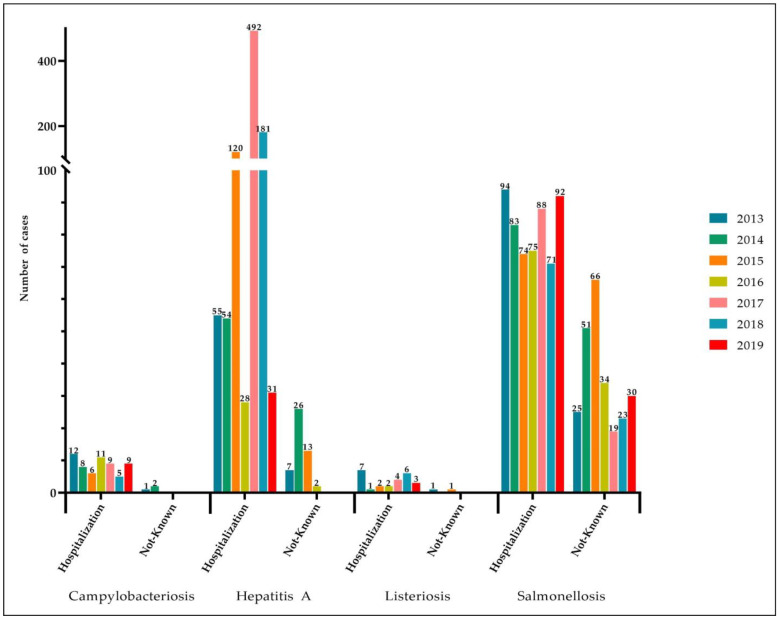
Number of hospitalized patients with campylobacteriosis, hepatitis A, listeriosis, and salmonellosis per year.

**Figure 2:**
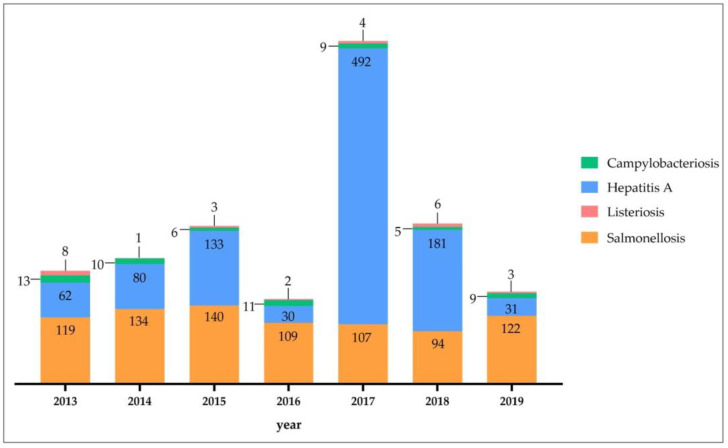
Distribution of confirmed cases of campylobacteriosis, hepatitis A, listeriosis, and salmonellosis by year.

In the Campania region, during the period 2013–2019, the total number of confirmed human cases of campylobacteriosis, hepatitis A, listeriosis, and salmonellosis was 1924. A total of 1624 (84.36%) people were hospitalized (Figure 1). Of the confirmed cases, 1116 (58.0%) were in females and 808 (42.0%) in males. The most affected age group was those within the range 25–44 (639, 33.21%). Napoli (990 cases), followed by Avellino (400), were the provinces in the Campania region with the highest number of cases. The highest number of confirmed cases was observed in 2017 (612) (Figure 2). Eliminating the influence of the high prevalence in 2017, the highest number of confirmed human cases of campylobacteriosis, hepatitis A, listeriosis, and salmonellosis was observed during the period 2013–2015 compared with the period 2016–2019.

### 3.1. Campylobacteriosis

**Figure 3:**
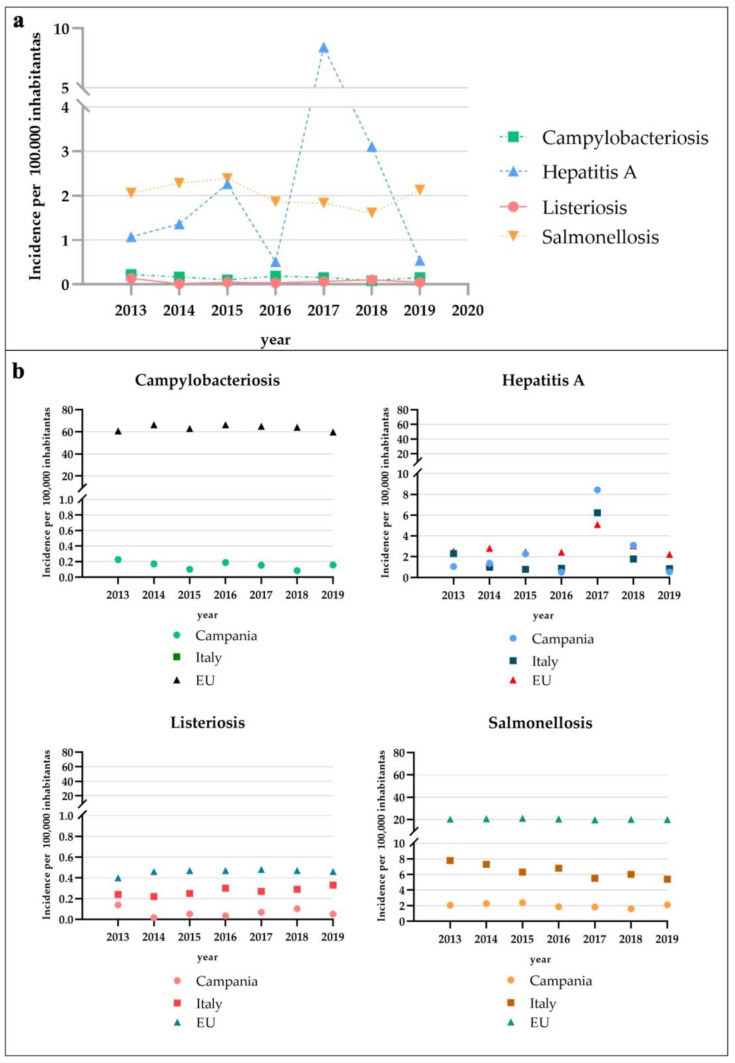
(**a**) Incidence of campylobacteriosis, hepatitis A, listeriosis, and salmonellosis, measured as the number of new cases of disease per 100,000 individuals in Campania (calculated on the whole population residing in the Campania Region between 2013 and 2019, https://demo.istat.it, accessed on 21 April 2022). (**b**) Comparison between the incidence data of Campania Region with those reported by the EFSA/ECDC (http://atlas.ecdc.europa.eu/public/index.aspx, accessed on 16 March 2022) in Italy and EU by year (2013–2019).

**Figure 4:**
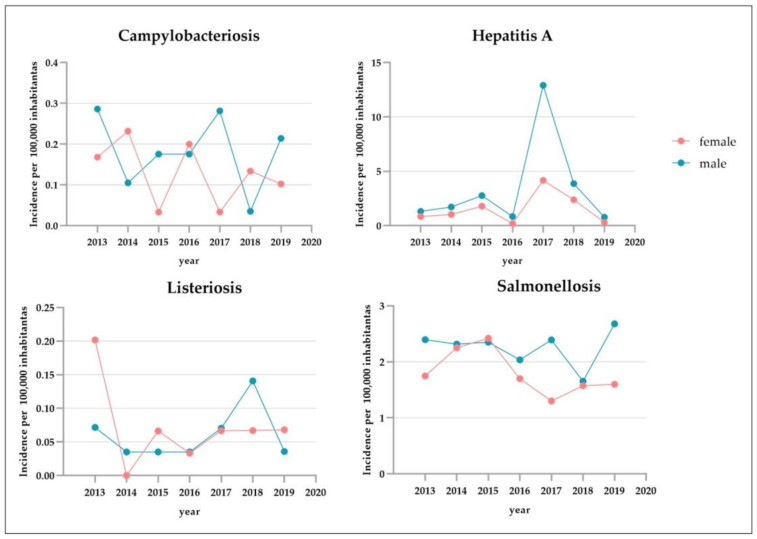
Incidence of campylobacteriosis, hepatitis A, listeriosis, and salmonellosis, measured as the number of new cases of disease per 100,000 female (pink line) and male (blue line) individuals in Campania (calculated on the female and male population residing in the Campania Region between 2013 and 2019, https://demo.istat.it, accessed on 21 April 2022) by year.

**Figure 5:**
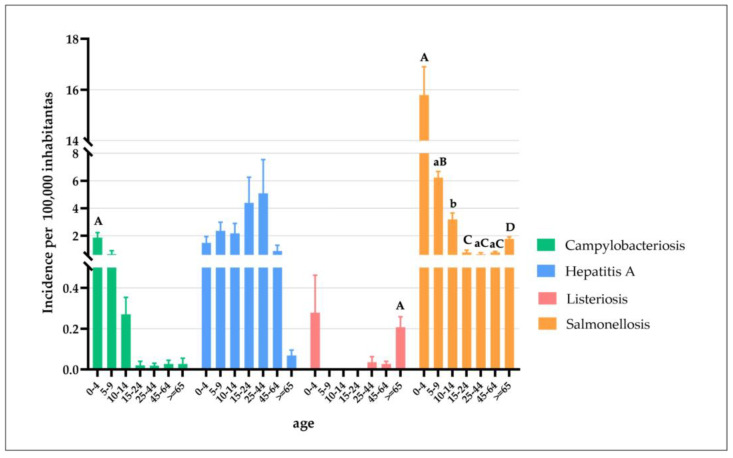
Mean annual incidence of campylobacteriosis, hepatitis A, listeriosis, and salmonellosis, measured as the number of new cases of disease per 100,000 individuals in Campania, by age and from 2013 to 2019. The mean age-specific incidence was calculated on the number of individuals per each age group (0–4 years, 5–14, 15–24, 25–44, 45–64, and 65 years and older) residing in the Campania Region between 2013 and 2019 (https://demo.istat.it, accessed on 21 April 2022). Statistical analysis was performed comparing the age-group with each other. Error bars represent the standard error of the mean. Different superscript uppercase letters indicate a significant difference at *p* < 0.01. Different superscript lowercase letters indicate a significant difference at *p* < 0.05.

**Figure 6:**
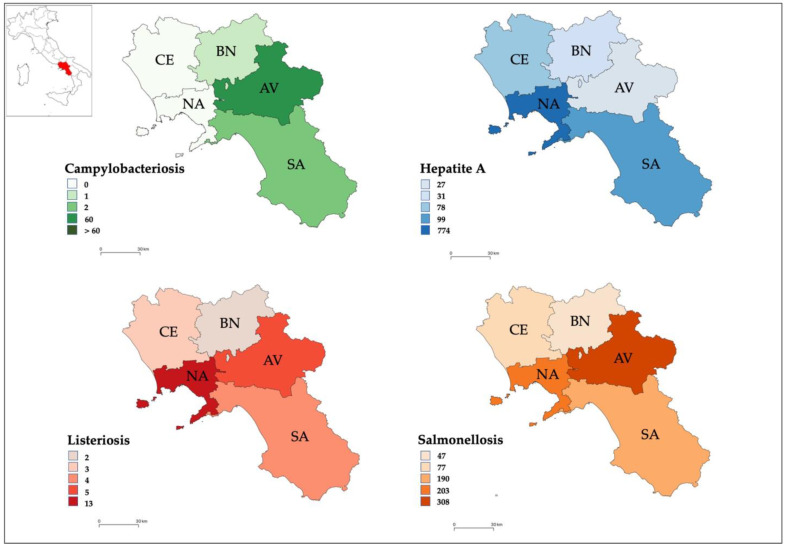
Geographical distribution in the Campania region (southern Italy) of disease notifications, underlining the prevalence in each province: Avellino (AV), Benevento (BN), Caserta (CE), Napoli (NA) and Salerno (SA). The results are expressed as the total number of confirmed cases in each province from 2013 to 2019.

During the period 2013–2019, the total number of confirmed human cases of campylobacteriosis was 63, with the highest incidence observed in 2013 (0.23 cases per 100,000 individuals) (Figure 3a). A total of 60 (95.24%) people were hospitalized (Figure 1). Of confirmed cases, 36 (57.14%, mean annual incidence: 0.13 per 100,000 individuals) were in females and 27 (42.86%, mean annual incidence: 0.18 per 100,000 individuals) in males (Figure 4). The highest number was detected among children under 5 years of age (n. 35, 55.56%, mean annual incidence: 1.86 per 100,000 individuals) (Figure 5). Avellino was the Province in the Campania region with the highest number of cases (Figure 6). Significant differences were found among age groups, and provinces (*p* < 0.01).

### 3.2. Hepatitis A

During the period 2013–2019, the total number of confirmed human cases of Hepatitis A was 1009 with the highest incidence observed in 2017 (8.43 cases per 100,000 individuals) (Figure 3a). During the years 2014, 2015, 2017, and 2018 the number of new cases observed in the Campania region was higher than those reported at the national level (Figure 3b). During the period 2013–2019, a total of 961 (95.24%) people were hospitalized (Figure 1). Of confirmed cases, 688 (68.19%, mean annual incidence: 1.53 per 100,000 individuals) were in females and 321 (31.81%, mean annual incidence: 3.46 per 100,000 individuals) in males (Figure 4). The most affected age group was those within the range 25–44 (n. 562, 55.70%, mean annual incidence: 5.09 per 100,000 individuals) (Figure 5). Napoli (n. 774 cases) was the Province in the Campania region with the highest number of cases (Figure 6). However, the observed differences (between year, sex, and age group) were not significant (*p* > 0.05), except for the territorial distribution of HAP infections (provinces; *p* < 0.01).

### 3.3. Listeriosis

During the period 2013–2019, the total number of confirmed human cases of Listeriosis was 27 with the highest incidence observed in 2013 (0.14 cases per 100,000 individuals) (Figure 3a). Throughout the period considered (2013–2019), the number of new cases of listeriosis reported in the Campania region was lower than those reported at the national level (*p* > 0.05) (Figure 3b). A total of 25 (92.59%) people were hospitalized (Figure 1). Of confirmed cases, 15 (55.56%, mean annual incidence: 0.07 per 100,000 individuals) were in females and 12 (44.44%, mean annual incidence: 0.06 per 100,000 individuals) in males (Figure 4). The highest number was detected among children under 5 years of age (5, 18.52%, mean annual incidence: 0.28 per 100,000 individuals) and people over 65 years of age (15, 55.56%, mean annual incidence: 0.21 per 100,000 individuals) (Figure 5). Napoli (13 cases) was the Province in Campania region with the highest number of cases (Figure 6). However, the observed differences (between year, sex, and provinces) were not significant (*p* > 0.05), except for the distribution of listeriosis among age groups (over 65 years; *p* < 0.01).

### 3.4. Salmonellosis

During the period 2013–2019, the total number of confirmed human cases of Salmonellosis was 825 with the highest incidence observed in 2015 (2.39 cases per 100,000 individuals) (Figure 3a). Throughout the period considered (2013–2019), the number of new cases of salmonellosis reported in the Campania region was lower than those reported at the national level (Figure 3b). A total of 577 (69.94%) people were hospitalized (Figure 1). Of confirmed cases, 377 (45.70%, mean annual incidence: 1.80 per 100,000 individuals) were in females and 448 (54.30%, mean annual incidence: 2.26 per 100,000 individuals) in males (*p* < 0.05) (Figure 4). The highest number was detected among children under 5 years of age (n. 395, 47.88%, mean annual incidence: 15.79 per 100,000 individuals) (Figure 5) (*p* < 0.05). Avellino (n. 308 cases) was the Province in the Campania region with the highest number of cases (Figure 6). The highest number of confirmed cases was observed in 2015 (140) (Figure 2). However, the observed differences in provinces were not significant (*p* > 0.05).

## 4. Discussion

This study focuses on the analysis of four gastrointestinal diseases (GI) in the Campania region over seven years, from 2013 to 2019. Based on the analysis of data on reportable diseases, an overall decline in the number of cases reported for campylobacteriosis, hepatitis A, listeriosis, and salmonellosis was detected. In particular, the annual incidence rates of gastrointestinal cases referred to the aforementioned infections ranged from 0.33 to 0.28 per 100,000 persons per year (2013–2019). However, it is worth highlighting that the homogeneity of the trend was interrupted by an anomalous peak (incidence: 1.01 per 100,000 individuals) in 2017, as a consequence of the high incidence of Hepatitis A cases reported in the year. Hepatitis A, as well as salmonellosis, was found to be the main cause of the incidence values of human GI cases in Campania. These findings are in agreement with several authors [12,13] who described the burden and the frequency of the hepatitis A virus in Campania from 1997 to 2018. To our knowledge, this is the first study that pays close attention to campylobacteriosis, salmonellosis, and listeriosis human cases in the Campania region; therefore, a direct comparison with preceding years is complicated. 

Interesting, in the Campania region reported cases of campylobacteriosis, listeriosis, and salmonellosis were found to be always lower if compared to Italian and European scenarios in the period from 2013 to 2019 (Figure 3b). This finding seems to suggest an appreciable efficiency of the regional health planning [14] in monitoring the spread of pathogenic microorganisms through foods vehicles. The role of the animal-origin foods in the transmission of these specific pathogens, and the human health implications of inefficient control plans, is well known [15]. However, it is impossible to ignore the likely underestimation of foodborne disease that could drastically shape the data. In keeping with the above, the hepatitis A case deserves a separate discussion due to the influence of several periodic strong outbreaks on the calculation of the incidence rates [13]. Indeed, in the year 2017, the regional incidence rate exceeded the National and European ones, with a difference of 2.21 and 3.33 (annual incidence rate per 100,000 individuals), respectively. 

### 4.1. Campylobacteriosis

For a long time, campylobacteriosis has been the most reported zoonosis in the European Union [16]. In the EU, the notification of campylobacteriosis is mandatory for most member states (MS); however, in Italy, as well as in Belgium, France, Greece, and Netherlands, *Campylobacter* infections are subject to voluntary notification. As regards the surveillance of campylobacteriosis, the surveillance systems cover the entire EU population except for four MS, including Italy [16]. In Italy, the Istituto Superiore di Sanità (Rome, Italy) coordinates the sentinel surveillance system, called Enter-Net Italia, that collects data and strains of campylobacter human cases voluntarily reported (covering about 65% of the Italian territory [17]), as well as others enteric bacterial pathogens such as *Salmonella*, and *Listeria*. So far, the Ministerial Decree (DM) 15/12/1990 does not include *Campylobacter* spp. among the agents subject to mandatory notification; however, in the PREMAL platform the item campylobacteriosis is present. Until this gap is resolved it is not possible to know the true burden of all those enteric pathogens which should currently be reported as “non-salmonella infectious diarrhea”.

According to our epidemiological data, *Campylobacter* spp. infections were infrequent in the Campania region in the period from 2013 to 2019. This assessment is based on the significant differences pointed out between campylobacteriosis incidence records in Campania and the EU (Figure 3b). However, the particularity of the Italian surveillance system and the consequence of the voluntary system of notifications on the exactitude of the number of human campylobacteriosis cases cannot be ignored. Indeed, several authors [18,19] agree on the concrete risk of underestimating the burden of *Campylobacter* spp. in Italy. 

To correctly analyse the Campania incidence rates of campylobacteriosis, our records were compared with those from the analysis of Enter-Net Italia database carried out by García-Fernández et al. [20] on the Italian territory. In particular, the authors analysed a smaller period, from January 2013 to December 2016, and recorded 4626 confirmed campylobacteriosis cases in the Italian country. These data are particularly interesting if the mean annual incidence rate is calculated and compared with those of the Campania region in the same years. Despite the same sentinel surveillance system, the study showed a clear prevalence of human cases outside the Campania region, demonstrated by the huge difference between the Italian and Campania incidence values, which were found to be 79.24 and 0.69 per 100,000 individuals, respectively.

As far as gender is concerned, females accounted for more cases of campylobacteriosis (no significant differences were found) (Figure 4), although this finding is not in agreement with several authors who have described a significant prevalence in the male gender in Italy [20], as well as in other countries of the world [21,22]. It is difficult to explain this unexpected result, especially if we consider that no clear explanations have been found to justify the predilection towards males [23]. However, even if no significant differences were found regarding gender in this study, it is possible to assume that a crucial role in determining gender sensitivity is played by typically sex and age-oriented tasks and dietary habits that might differ in each country/region [24]. Regarding the distribution of human cases by year, a significant correlation with age (*p* < 0.01) was highlighted, and 0–4 years old children were depicted as the most sensitive population (Figure 5), consistent with the scientific literature [20,21]. 

Although no data are available to prove the responsibility of food as the main responsible for these confirmed human cases in Campania, a deeper analysis of the literature could help to analyse the potential links between contaminated food and human infections. For example, recently Kerkhof et al. [25] pointed out an important circulation of the bacterium among wild boars hunted in the Campania region (50% of fecal samples were found positive for *Campylobacter* spp.) and the alarming role of wild boars as reservoirs of the pathogen. These findings are consistent with those of Peruzy et al. [26], who carried out an evaluation of the microbial contamination of 36 eviscerated carcasses of wild boars collected in Campania region in the hunting period from October to December 2019. In this study, the occurrence of *Campylobacter* spp. was found around 11%. Obviously, these data, as well as the others present in literature, are not sufficient to assume that food has played a crucial role in the onset of human cases of campylobacteriosis in Campania. However, it is worth taking into mind the evidence and not forgetting the key role of foods as carrier of pathogenic bacteria as reported by several authors and European reports [15,16,24,27]. 

### 4.2. Hepatitis A

The hepatitis A virus (HAV) is responsible for numerous viral hepatitis cases in the world, recording the highest prevalence rates in developing countries (Central and South America, Asia, and Africa) mainly due to poor hygienic conditions [28] that contribute to the widespread of pathogens through contaminated foods and water, and person-to-person contacts. Otherwise, in developed countries, as well as in Italy, hepatitis A is lowly/intermediately endemic, and the appearance of new cases has been attributed to sporadic outbreaks in adults [29]. In particular, in the last decades in the Campania region, the annual incidence rate related to HAV was influenced by periodic outbreaks [12].

In Italy, hepatitis A is a notifiable enteric illness. The surveillance of hepatitis is based on the notifications of the PREMAL System integrated with information from the questionnaires of SEIEVA, which is managed by the Istituto Superiore di Sanità (ISS). Although the SEIEVA system ensures a deeper knowledge of epidemiology, through the comprehension and the evaluation of the relative contribution of the various associated risk factors, this surveillance system makes use of the network of Local Health Units (LHUs) that participate voluntarily. Considering this, it may be assumed that the same information is not entered in both systems, and inequality between PREMAL and SEIEVA databases may exist. For instance, in the year 2017, 46 human cases of hepatitis A were recorded in the SEIEVA surveillance system (https://www.epicentro.iss.it/epatite, accessed on 22 April 2022); however, these data were inconsistent with those of the PREMAL database where the value in 2017 was ten times higher. The inconsistency of the surveillance systems adopted in Italy could cause confusion and bad information among the population, as well as among scientists [30]. Therefore, the comparison of data in the present work with those of several authors [12,13] is complicated, due to analyses on the SEIEVA dataset.

In accordance with our findings, in 2015, 2017, and 2018, the incidence rates of cases of hepatitis A in the Campania region were found to be higher than those recorded in Italy. Due to the lack of information about the source of contamination, a task force was set up in Italy in 2015 [13] to extend and combine surveillance systems, matching results of humans, foods, and environmental isolates. The role of contaminated water and bivalve shellfish as carriers of the virus is well known and described [31,32], so much so that the HAV outbreak that occurred in 2015 in Campania region was limited by monitoring bivalve shellfish as the major source [12]. Although strict control measures have been adopted on the bivalve supply chain, in the same Neapolitan area a new peak of infection was reached after two years. In this regard, according to La Rosa et al. [13] the variants identified in 2017–2018 were different from those mainly detected in the previous outbreak (2015), the main source being two new HAV variants occurring in a multistate outbreak affecting mostly men who have sex with men (MSM) [33]. Even in these years, shellfish and water were the main vectors of the virus in the Campania region [13]. These findings justify the prevalence of HAV-reported human cases along the regional seacoast, especially in Naples (*p* < 0.01) where the consumption of bivalve shellfish is a cultural and religious tradition [34].

Due to the low endemicity of the Hepatic Virus A in Italy, infections are usually found in susceptible adults who may manifest severe symptoms [35]. The rate of hospitalization in this country has been extensively described by Severi et al. [36] for the years 2001–2013; the authors were surprised by the records of hospitalizations that exceeded those of the notifications, and they explained this paradox considering the well-known underestimation of HAV human cases in Italy, as reported previously [30]. According to PREMAL data relating to hospitalization rates during the period 2013–2019, in Campania about 95% of all individuals registered in the surveillance system needed hospitalization. This finding confirmed the severity of HAV infections in the case of low-endemic countries, such as Italy.

Regarding gender, although females accounted for most cases of hepatitis A, no significant sex differences were found. Moreover, the virus seems to prefer adult individuals in sporadic outbreaks that occurred in Campania, but the differences between age groups were not significant (*p* > 0.05); however, other authors described an age specificity referred to people aged between 5 and 15 years [21].

### 4.3. Listeriosis

Listeriosis is a foodborne disease characterized by low incidence rates among developed countries [37]. Despite this, the responsible bacterium is capable of severely affecting susceptible individuals, accounting for the highest hospitalization and mortality rates among other zoonoses [15,16]. Since 2008, surveillance by the European Union (EU) and the European Economic Area (EEA) has focused on invasive forms of listeriosis. In Italy, listeriosis is a foodborne illness subject to mandatory notification, included among relevant second-class diseases according to the Ministerial Decree of 15 December 1990. Furthermore, in 2010, listeriosis was added to the surveillance network of Enter-Net Italia. Although for most infectious diseases (e.g., campylobacteriosis and salmonellosis) the total number of reported cases represents a small part of total cases, the so-called “tip of the iceberg” [17,37,38], the underestimation of invasive listeriosis could be low due to the severity of symptoms that reduce the phenomena of underdiagnosis and/or under-reporting. However, the incubation period (from a couple of days to two weeks) of invasive listeriosis, as well as the likely number of asymptomatic people, makes it difficult to identify infectious vehicles. Therefore, national mandatory notification needs integration with a molecular investigation to better analyse the epidemiology of this foodborne illness, differentiating sporadic cases from strong foodborne outbreaks. To this end, and in accordance with the objective of European surveillance for the investigation of the molecular epidemiology of human listeriosis, Italy participates in the ELiTE-WGS (European *Listeria* Typing Exercise—Whole Genome Sequence) project which aimed to integrate the EFSA-ECDC joint database with the data obtained through the WGS analysis of isolates and the collaboration of the various Competent Authorities. In Italy, the Istituto Superiore di Sanità (ISS) and the National Reference Laboratory for *Listeria monocytogenes* deal with this activity.

Although it is well known that the achievement of accuracy of epidemiological data of listeriosis in Italy is still a long way off [39], this study investigated the records of the PREMAL mandatory notification system in the Campania region in the period 2013–2019. According to our analysis, the epidemiological scenario in Campania reveals a very low frequency of serious illness, especially when compared with those of Italy and the European Union in the same years (Figure 3b). It is worth considering that the underestimation of listeriosis human cases could influence the trend of incidence and the truthfulness of the data.

In Campania, the incidence rates varied depending on the years, ranging between 0.14 and 0.02 per 100,000 inhabitants. In this regard, no epidemic events were reported for 2013, the year in which the highest value was reached. In more detail, no significant differences were found even between the five provinces, so much so that the mean annual number of human cases ranged from 2.17 to 1 in Naples, and Caserta and Benevento, respectively.

As expected, the number of hospitalized people was very close to that of the total number of reported human cases of listeriosis. These data are consistent with the literature [37,40] and with the latest report of EFSA/ECDC which defines listeriosis as the zoonosis with the highest proportion of hospitalizations among those under EU surveillance [16]. Indeed, de Noordhout et al. [40] assessed the burden of listeriosis, aware of the lack of information in the literature, and estimated that it caused 11,132 DALYs (95% CrI 8656-13,991) in 2010 in the WHO EURO A subregions. The high sensitivity of at-risk groups of people towards *Listeria monocytogenes* could explain the serious burden of this severe illness compared to other foodborne diseases. Infants, pregnant women, the immune-compromised, and elderly people are the main subjects affected by severe forms of listeriosis. As shown in Figure 5, in Campania the elderly and infants (over 65 and 0–4 years, respectively) were the most affected. In particular, significant differences were found between the elderly group and others (*p* < 0.01), confirming old age among factors that most influence the sensitivity of the population to the bacterial pathogen. However, although pregnant women are included among the sensitive people, no significant sex differences have been found for listeriosis in Campania. This finding is consistent with Zolin et al. [37] who pointed out a low number of pregnancy-related reported cases likely due to the underdiagnosis of fatal pregnancies attributable to listeriosis. For this reason, it is possible to hypothesize a similar scenario also in the Campania region.

### 4.4. Salmonellosis

Nontyphoidal *Salmonella* infections (NTS) are the second most reported zoonoses and the first cause of confirmed foodborne outbreaks in the EU. In the EU, the notification of NTS is mandatory in almost all member states, including Italy, and the EU surveillance system covers the entire population, except France, Netherlands, and Spain [16]. In more detail, the Italian notification system for salmonellosis is based on the DM 15 December 1990 according to which notification is mandatory by producing a report within 48 h of observing the case. As reported above for campylobacteriosis and listeriosis, the PREMAL notification system is complemented by a passive laboratory-based surveillance system for enteropathogens, Enter-Net Italia, which collects microbiological information on *Salmonella* strains isolated from human patients. However, it is estimated that only about 50% of all reported human cases have a matching strain entered into the Enter-Net database [41]. Mascaro et al. [42] highlighted the differences in sensitivity of surveillance systems among the Italian regions, due to the non-homogeneous distribution of designated laboratories on the national territory. In particular, Graziani et al. [17] considered the differences in diagnostic capacity among laboratories to justify the low incidence rates of *Salmonella* isolates in southern Italy. Unfortunately, the level of under-reported cases of salmonellosis is still high, and the lack of useful information on the main isolates undermines the approach of physicians who could be guided in choosing the right pharmacological treatment.

In this study, the incidence rates of salmonellosis in the Campania region (2013–2019) were calculated using data from the PREMAL notification system. Although Campania has been considered one of the Italian regions with the lowest incidence rates referred to salmonellosis in the last three decades [17], the data processing was based on the awareness of the existence of weak spots in the national notification system, mainly due to the underdiagnosis of asymptomatic forms of salmonellosis.

In the period from 2013 to 2019, the annual incidence rates of salmonellosis in the Campania region were always found to be lower when compared to those of Italy and the EU. Interestingly, the regional trend remained constant throughout the period and diverged significantly from that published by EFSA for salmonellosis in EU, with a tenfold difference between them. Although the data should suggest a low salmonellosis burden in Campania, the percentage of people hospitalized exceeded that in the EU in the same years. For instance, in the EU only 42.5% of all confirmed cases with hospitalization information belonged to the hospitalized proportion in 2018; however, in the same year, the percentage of hospitalized people in Campania was 75.41%. It is worth noting that this proportion remained almost constant during the entire period of study, with annual percentages ranging from 52.86 to 82.24 in 2015 and 2017, respectively. The data suggest two different hypotheses: (1) in the Campania region, salmonellosis human cases are caused by very pathogenic *Salmonella* serotypes, and this could explain the prevalence of severe forms; (2) in the Campania region, underdiagnosis drastically affects the calculation of the incidence rates, hiding the problem. This situation could be resolved by stepping up the laboratory-based surveillance system, and recording useful information to reconstruct the epidemiologic scenario of each case of infection.

Regarding people’s gender in reported cases of salmonellosis, differences in incidence rates emerged between males and females in Campania, contrary to that reported by Graziani et al. [17] for Italy. However, data on demographic distributions were found to be consistent with the literature [17,42] that considered younger children (0–4) to be the most affected, probably due to the high frequency of severe forms among them.

As regards the potential role of foods as the vehicle, the data reported in the Enter-vet (Enter-vet Network, coordinated by the National Reference Center for Salmonellosis of the IZS delle Venezie, which aims to collect data at national level relating to the isolation of *Salmonella* spp., from samples of veterinary origin) reports (from 2013 to 2019, https://www.izsvenezie.it/temi/malattie-patogeni/salmonella/enter-vet/, accessed on 4 September 2022) as well as in the literature suggests that a link between humans and food isolates of *Salmonella* could be hypothesized. For instance, Peruzy et al. [43] reported, for the period under study, an important and growing number of *Salmonella* isolates in foods and carcasses in Campania and Calabria regions, ranging from 42 (3.06%) to 113 (12.46%) in the years 2013 and 2019, respectively. Although the authors did not show region-specific data for year, approximately 74% (73.93% and 74.41% for the sampling periods 2011–2015 and 2016–2021) of all positive samples analysed in the study were collected in Campania [43]. The authors explained these findings by the high animal number and the livestock density in the region. Our results with those cited above suggest that the bacterium is present and circulates in the regional territory, and foods are probably involved in the onset of salmonellosis infection in humans.

## 5. Conclusions

In conclusion, epidemiological surveillance of gastroenteritis is crucial in infection control, and helps to orient prevention and control campaigns. During the seven years under investigation (2013–2019), an overall decline in the number of cases reported for campylobacteriosis, hepatitis A, listeriosis, and salmonellosis was detected. Unlike hepatitis A, the incidences of campylobacteriosis, listeriosis, and salmonellosis were found to be always lower compared to European trends. However, surveillance systems that allow monitoring the incidence, trends, and spatiotemporal burden of care of infections, and detecting outbreaks and their origins throughout the food chain, are indispensable. The surveillance system currently available in Campania does not include all these features and, therefore, they should be implemented.

## Data Availability

The datasets used and analysed in this study are available from the corresponding author upon reasonable request.
